# An ecological approach to monitor geographic disparities in cancer outcomes

**DOI:** 10.1371/journal.pone.0218712

**Published:** 2019-06-21

**Authors:** Jinani Jayasekera, Eberechukwu Onukwugha, Christopher Cadham, Donna Harrington, Sarah Tom, Francoise Pradel, Michael Naslund

**Affiliations:** 1 Lombardi Comprehensive Cancer Center, Georgetown University Medical Center, Washington, DC, United States of America; 2 Department of Pharmaceutical Health Services Research, School of Pharmacy, University of Maryland, Baltimore, MD, United States of America; 3 School of Social Work, University of Maryland, Baltimore, MD, United States of America; 4 Division of Neurology Clinical Outcomes Research and Population Science (NeuroCORPS), Department of Neurology, Columbia University, New York, NY, United States of America; 5 School of Medicine, University of Maryland, Baltimore, MD, United States of America; University of North Texas Health Science Center, UNITED STATES

## Abstract

**Background:**

Area-level indices are widely used to assess the impact of socio-environmental characteristics on cancer outcomes. While area-level measures of socioeconomic status (SES) have been previously used in cancer settings, fewer studies have focused on evaluating the impact of area-level health services supply (HSS) characteristics on cancer outcomes. Moreover, there is significant variation in the methods and constructs used to create area-level indices.

**Methods:**

In this study, we introduced a psychometrically-induced, reproducible approach to develop area-level HSS and SES indices. We assessed the utility of these indices in detecting the effects of area-level characteristics on prostate, breast, and lung cancer incidence and stage at diagnosis in the US. The information on county-level SES and HSS characteristics were extracted from US Census, County Business Patterns data and Area Health Resource Files. The Surveillance, Epidemiology, and End Results database was used to identify individuals diagnosed with cancer from 2010 to 2012. SES and HSS indices were developed and linked to 3-year age-adjusted cancer incidence rates. SES and HSS indices empirically summarized the level of employment, education, poverty and income, and the availability of health care facilities and health professionals within counties.

**Results:**

SES and HSS models demonstrated good fit (TLI = 0.98 and 0.96, respectively) and internal consistency (alpha = 0.85 and 0.95, respectively). Increasing SES and HSS were associated with increasing prostate and breast cancer and decreasing lung cancer incidence rates. The results varied by stage at diagnosis and race.

**Conclusion:**

Composite county-level measures of SES and HSS were effective in ranking counties and detecting gradients in cancer incidence and stage at diagnosis. Thus, these measures provide valuable tools for monitoring geographic disparities in cancer outcomes.

## Background

Research has found that a narrow focus on individuals outside of their social and physical contexts limits our understanding of disease etiology, outcomes, and interventions [1–3]. Socioeconomic forces that determine demand for health care and market forces that determine health services supply (HSS) may influence cancer outcomes directly or indirectly through individual characteristics [4]. These contextual effects lead to social patterning of cancer outcomes through area-level stratification processes that allow individuals to obtain health-enhancing knowledge, and take advantage of this through prevention, screening, and early detection.

Historically, researchers have used both single measures representing contextual attributes (e.g. percentage below poverty level), as well as composite indices consisting of several attributes to examine the impact of area-level characteristics on cancer outcomes [5–10]. A composite index of key indicators has the ability to reflect the multidimensional nature of a community’s socioeconomic status (SES) more accurately than single measures of area-level characteristics [5, 11, 12]. In addition, area-level indicators (e.g. income, poverty, and occupation) tend to be highly correlated, which may lead to multicollinearity in a multivariable analysis [13, 14]. Therefore, a composite index should have greater validity, robustness, and explanatory power than single area-level measures in documenting the impact of area-level characteristics on disease outcomes [5, 14]. However, research addressing area-level composite indices have paid limited attention to psychometric techniques that can be used to develop these measures. As a result, the rich tradition of psychometrics has not been fully exploited in the development and testing of area-level indices [2]. Limited focus on the application of standard psychometric techniques in the construction of area-level indices has contributed to a lack of consistency in variables used to create these measures, and comparability across measures [2, 12]. Composite measures of SES have included different combinations of occupation, employment, poverty, income, education, housing, ownership and living crowdedness variables, thus, limiting the ability to systematically compare and assess the impact of area-level SES on disease outcomes [12].

Prostate, breast, and lung and bronchus cancer are the three most frequently diagnosed, and the leading causes of cancer mortality in the US and worldwide [15, 16]. Globally, there is pronounced geographic variation in incidence rates across these three cancer sites [17–21]. The risk of these cancers is susceptible to human intervention through screening, early-detection, and prevention [22–24]. Thus, geographic variation in prostate, breast, and lung and bronchus cancer incidence rates has been attributed to differences in the use of screening tests and diagnostic practices, prevalence of risk factors such as smoking and obesity, as well as disparities in the distribution of socioeconomic characteristics and access to health care both in the US and worldwide [17, 21, 25, 26].

Area-based composite indices of SES have been used extensively in cancer settings in the US. However, fewer studies have explored the aggregate impact of HSS characteristics including a broad range of facilities, services, and physician/non-physician health care providers on the incidence of cancer. The availability of health care services has the potential to impact cancer incidence through health-seeking behavior, access to health-promoting resources, travel distance, crowding, waiting times, and patient-provider relationships. Measures capturing health care resource scarcity may provide useful tools for identifying areas to which resources could be allocated to improve delivery of health services and potentially reduce geographic disparities in cancer incidence [27].

In the current study, we focused on introducing a psychometrically-induced, reproducible approach to developing county-level indices to capture area-level SES and HSS characteristics. Using these composite measures, we examined the effects of county-level SES and HSS gradients on prostate, breast, and lung and bronchus cancer incidence and stage at diagnosis in the US.

## Methods

### Geographic unit

No general consensus exists regarding the geographic level at which area-based measures should be developed in the US [7]. SES indices have been developed at several levels using block, census tract, zip code, and county-level data [6–10, 14, 22, 28, 29]. Previous studies have used smaller areas, such as census tracts and blocks due to the likelihood of homogeneity in population characteristics, economic status, and living conditions [5, 6, 13]. While blocks and census tracts are more likely to be socioeconomically homogeneous than larger geographic units, they are more susceptible to change over time [5]. Zip codes, were established by the US Postal Service for efficient delivery of mail [7], and they do not provide a meaningful basis for economic or health services planning [30].

Counties are legislative areas with 100,000 persons on average, and are socio-politically and geographically more stable than census tracts and blocks [5, 13]. Counties provide an appropriate socioeconomic, political, and community context within which many social and public health policies are formulated and implemented in the US [5, 31, 32].

### Data sources

We used the 2006–2010 American Community Survey (ACS) 5-Year estimates to extract county-level socioeconomic characteristics. Annual County Business Patterns (CBP) data for 2010 was used to obtain information on the availability of health care facilities and services [33]. Area Health Resource Files (AHRF) were used to obtain information on the availability of health care professionals in 2010. Data on cancer incidence and stage at diagnosis from 2010 to 2012 were collected using the National Cancer Institute’s (NCI) Surveillance, Epidemiology, and End Results (SEER) Public Use Database [34]. Each of SEER’s 18 regional cancer registries collects data on patient demographic characteristics, primary tumor site, tumor morphology, stage at diagnosis, and first course of treatment on all diagnosed cancers within its region [34].

### County-level characteristics

The literature is replete with different SES indices that are composed of various combinations of socioeconomic indicators [3]. Particularly important to this analysis were the area-based composite SES indices developed by Singh [5, 28], Yost [6], Krieger [7], Dayal [8], Saldana-Ruiz [10], and Rubin [22], as these indices have shown associations with cancer incidence and/or mortality at different geographic levels. The combinations of various area-level characteristics used by these SES indices belong to the broad domains of occupation, employment, education, poverty, income, housing, ownership, and living crowdedness. For example, the ‘poverty’ domain included percentage of persons below poverty, multiples of the poverty line, and percentage of families below poverty-level. We extracted thirty county-level socioeconomic characteristics belonging to the eight domains from the 2006–2010 ACS.

All health care personnel, facilities, and services that could potentially influence the uptake of screening services and early detection of cancer were considered for HSS index development. The number of facilities or providers available within the county was divided by the total land area of each county, and then multiplied by 1,000 to express each health care characteristic as the number available per 1,000 square miles to capture health care resource availability given the size of the county.

### Statistical analysis

#### Index development

Structural equation modeling techniques were used to arrive at the number and nature of latent constructs needed to account for the correlations, and to capture the commonality among the variables. The analytic step was conducted using the SAS PROC Factor procedure with a maximum likelihood (ML) parameter estimator. We first tested the factor structures of the previously developed SES indices using county-level data extracted from the 2006–2010 ACS [5–8, 10, 22, 28]. After evaluating the psychometric properties of the existing indices, we created a new county-level SES index using a richer, psychometrically induced approach. Twenty-three indicators were selected for the new index based on the conceptual definitions of SES, and empirical evidence from the aforementioned studies highlighting the effects of area-level SES on cancer outcomes.

The creation of the new SES and HSS indices involved the following steps. All measures were normalized using rank transformations prior to being entered into ML factor analysis; tied values were assigned an average rank [13]. We used ML factor analysis with orthogonal rotations to simplify and clarify the data structure [35]. An orthogonal rotation was preferred to an oblique rotation as the factors were expected to be uncorrelated constructs [36]. The initial models were retained, based on scree plots and Kaiser criterion (i.e. Eigen value greater than 1.0) [37]. According to Costello [35], if an item has a factor loading of less than 0.40 it may either not be related to the other items, or it may suggest an additional factor that should be further explored. Cross-loaded items with values ≥0.40 on two or more factors were removed when other items had factor loadings of 0.50 or greater [35, 38]. We re-ran the model each time an item was deleted [38]. Factors which clearly indicated a theoretically and empirically meaningful clustering of the given indicators were retained. Factors with fewer than 3 items, with only a few substantial loadings which did not lend itself to any obvious theoretical interpretation were rejected [37]. The final models were chosen based on; 1) percentage of common variance explained (i.e. the proportion of the total variance accounted for by each factor), 2) achieving a “simple solution”, 3) interpretability of the factors, and 4) goodness-of-fit (TLI ≥0.95). Goodness-of-fit indices, such as the Tucker-Lewis Index (TLI), show how well a model fits data [39]. According to Hu and Bentler [40], the proposed TLI cut-off for an acceptable model fit in ML estimation is ≥0.95. Internal consistency, i.e. the degree to which responses are consistent across the items within a measure was tested using Cronbach’s alpha (α) [39]. In the final step, factor coefficients were used to construct weighted SES and HSS scores for each county.

#### Cancer incidence and stage at diagnosis

SEER data used to assess the gradients detected by SES and HSS composite measures included individuals at least 15 years of age, who lived in 611 counties covered by 17 SEER registries from 2010 to 2012. Unknown counties and Kalawao County in Hawaii were excluded due to lack of information on county-level characteristics. The county-level SES and HSS measures were merged with SEER data using the Federal Information and Processing Standards (FIPS) codes. FIPS codes recorded in SEER represent patient’s county of residence at the time of cancer diagnosis [13]. We examined the distribution of SES and HSS index scores across all counties (N = 3,138 and 3,143) and the counties covered by SEER (N = 611). SES and HSS scores were sorted from high to low, and divided as closely as possible into tertiles and quartiles with equal populations in each class based on their distribution across SEER county populations [13]. We examined the distribution of race/ethnicity and percentage living in urban/rural locations across the SES and HSS categories.

To analyze disparities in cancer incidence and stage at diagnosis by SES and HSS, we obtained 3-year incidence rates that were age-standardized to the 2000 US standard population and expressed per 100,000 population using NCI’s SEER*Stat software (version 8.2.1 released on April 2015) [41]. Incidence rates were calculated by stage at diagnosis using SEER historic staging information. We also examined the relationships between the county-level composite measures and cancer incidence separately among White, African American, and Asian/Pacific Islander race groups. American Indian and Alaska Natives were excluded due to small sample size. The trends in the gradients detected by SES and HSS indices were verified using a nonparametric trend test [42] to detect trends across ordered groups.

Analyses were performed using SAS software, version 9.2 (SAS Institute, Inc., Cary, NC, USA). SAS JMP software (11.2.0) was used to examine the distribution of the SES and HSS scores across the counties.

## Results

### County-level indices

**Fig 1:**
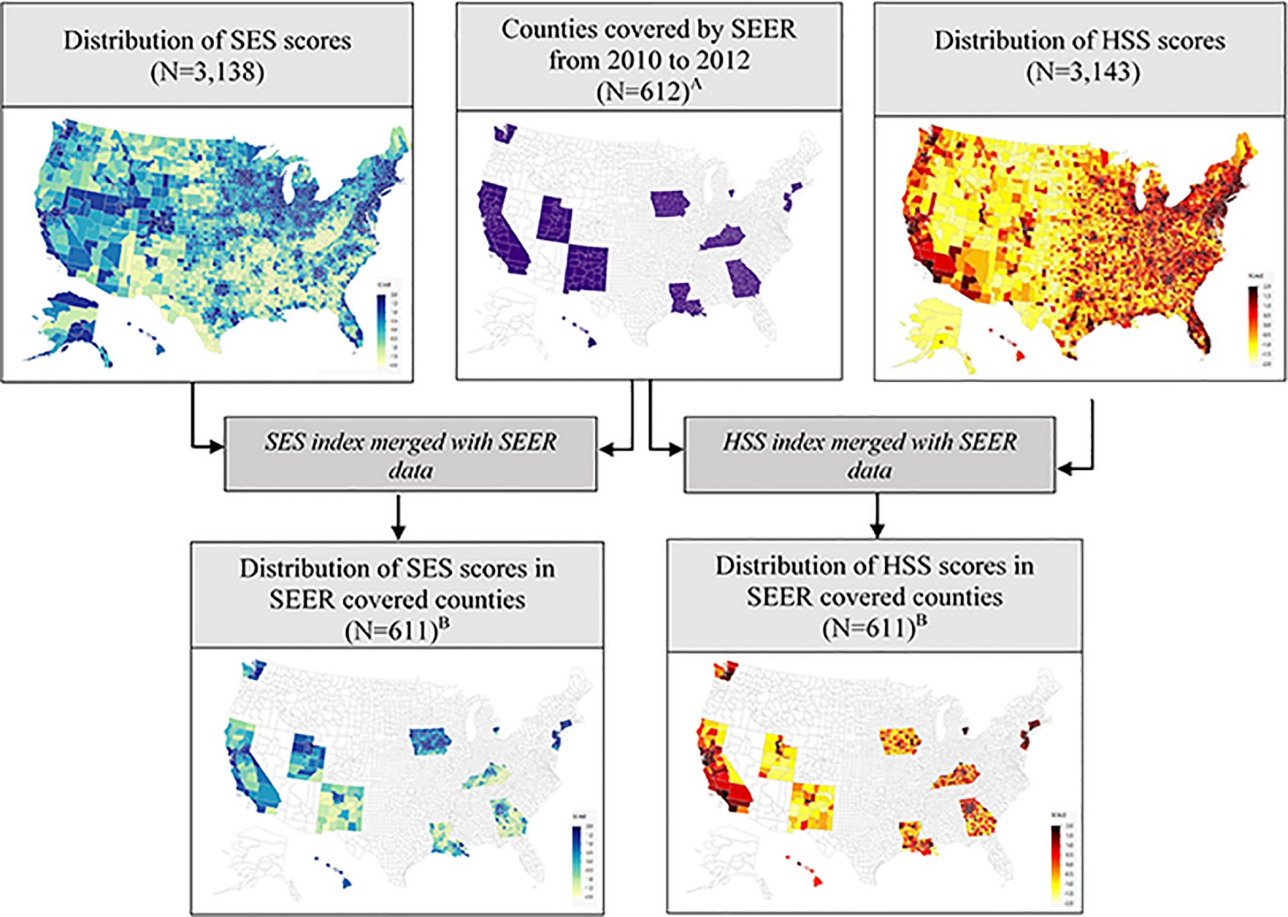
Flow diagram illustrating data availability and the distribution of SES and HSS index scores. In the maps showing the distribution of SES and HSS scores, the darker shades represent high SES and high HSS areas, and the lighter shades represent low SES and low HSS areas. Areas with no information are not shaded. A. SEER data extracted from 17 registries, excluding Alaska tumor registry. B. Kalawao County in Hawaii was excluded due to lack of SES data. *N* number of counties, *SEER* Surveillance, Epidemiology, and End Results program, *SES* Socioeconomic Status, *HSS* Health Services Supply.

The factor structures of the six indices tested [5–8, 10, 22, 28] varied with respect to the combinations of SES measures used, factor loadings, common variance explained, and goodness-of-fit (S1 Table). The new SES index developed using the candidate indicators retained three factors after rotation, with the first factor explaining 80% of the common variance (TLI = 0.79) (S2 Table). Fourteen of the indicators were clustered and had considerably larger loadings (≥0.40) on the first factor than on the second or third factor. Six SES indicators had cross loadings with values ≥0.40 on two factors. SES index development involved an iterative process of excluding cross-loaded items with values ≥0.40 on two or more factors, rejecting factors with fewer than 3 items, and retaining factors using multiple criteria including the Kaiser rule and scree plots [37]. The final single component model retained, employment rate, % of families below poverty level, median family income, and an education index [43] (weighted school years) (Table 1). The large absolute coefficients of poverty and income indicators suggest that they were dominant contributors to the SES index. The TLI value for the model created using the full sample (N = 3,138) was 0.98, indicating excellent model fit. The distribution of SES scores across the 3,138 counties ranged from -1.82 to 1.80, with lower values representing low SES, and higher values representing high SES (Mean: 0.00, SD: 0.96, Skewness tests: p-value = 0.92). The distributions of the SES scores across all 3,138 US counties and 611 SEER covered counties are shown in Fig 1. Majority of the relatively low SES counties were concentrated in the southwestern and southeastern regions of the US.

**Table 1:** Factor loadings and fit statistics of the new county-level composite SES and HSS indices.

**SES domain**	**County-level SES measures** ^**a**^	**Factor Loadings**
		**SES index** **(N = 3,138)**
Employed	Civilian labor force population aged ≥16 years employed, % (employment rate)	0.794
Poverty	Families below poverty level, %	- 0.872
Income	Median family income	0.926
Education	Education index (weighted school years)^b^	0.507
	*TLI*	*0*.*984*
	*Cronbach’s alpha (Standardized)*	*0*.*850*
**HSS domain**	**County-level HSS measures** ^**c**^	**HSS index** **(N = 3,143)**
Facilities	Physician offices	0.949
	Medical and Diagnostic Laboratories	0.742
	General Medical and Surgical Hospitals	0.735
Providers	Primary Care Practitioners ^d^	0.952
	Physician Assistants	0.800
	Registered Nurses	0.920
	Public Health and General Preventive Medicine Practitioners ^e^	0.550
	Pharmacists	0.903
	Diagnostic Radiology ^e^	0.776
	*TLI*	*0*.*962*
	*Cronbach’s alpha (Standardized)*	*0*.*947*

a: Extracted from the 2006–2010 American Community Survey 5-Year Estimates.

b: A weight of 16 was applied to the proportion of persons in the county with a college education (pc); 12 was applied to the proportion with a high school education (phs); and nine was applied to the proportion with less than a high school education (po). The average years of schooling in a given county, E = (16*pc) + (12*phs) + (9*po) [43]

c: Extracted from the 2010 Area Health Resource files and County Business Patterns data.

d: Primary Care Practitioners include non-Federal doctors of medicine and doctors of osteopathy providing direct patient care who practice principally in general or family practice and general internal medicine.

e: Subspecialty data extracted from the 2010 American Medical Association Physician Masterfiles.

*SES* Socioeconomic status, *HSS* Health services supply, *N* Number of counties, *TLI* Tucker-Lewis Index

Based on the Kaiser criterion and a scree plot, one component with a TLI of 0.96 was retained for the HSS index (Table 1). The composite measure combined 3 indicators of ‘facilities’ and 6 indicators of ‘providers’. The distribution of the HSS scores across 3,143 counties ranged from -1.70 to 1.86, with lower values representing low HSS, and higher values representing high HSS (Mean: 0.00, SD: 0.99, Skewness tests: p-value = 0.88). Majority of the relatively low HSS areas were located in rocky mountain and southwestern regions (Fig 1). Pearson’s correlation coefficient between SES and HSS measures was 0.29 (p<0.01), indicating a weak linear relationship between the two measures.

### Effects of SES and HSS gradients on cancer incidence and stage at diagnosis

In the 611 counties covered by SEER from 2010 to 2012, a total of 167,959 men aged 15 years and above were diagnosed with prostate cancer, of which 151,163 (90%) were diagnosed with incident localized/regional prostate cancer, and 8,398 (5%) with incident distant prostate cancer. Similarly, among 180,748 women diagnosed with breast cancer, 63% were with localized, 27% were with regional, and 7% were with distant breast cancer at diagnosis. Among 151,740 individuals diagnosed with lung and bronchus cancer, 20% were with localized, 23% were with regional, and 51% were with distant cancer at diagnosis. Counties belonging to the high SES group consisted of a lower proportion of African Americans and individuals living in rural areas compared to the lowest SES category (Table 2). Counties belonging to the high HSS group consisted of a higher proportion of African Americans and individuals living in urban areas compared to the lowest HSS category (Table 2).

**Table 2:** Distribution of county characteristics by SES and HSS classes, 2010–2012 SEER 17 combined (N = 611).

Index	Classification Scheme	Class	Population	Row Percent						
			Total	White	African American	American Indian	Asian	Hispanic	Urban ^a^	Rural ^b^
**SES**	Tertile	High	28,627,476	66.67	6.86	0.54	13.59	17.27	73.76	26.24
		Median	25,161,164	67.31	12.92	0.91	5.95	20.77	54.65	45.35
		Low	32,075,546	61.16	14.85	1.29	6.25	28.67	37.04	62.96
	Quartile	High	24,432,212	67.19	6.41	0.52	13.82	16.84	76.40	23.60
		2	18,196,224	69.89	10.29	0.76	7.40	17.38	59.99	40.01
		3	21,195,612	56.63	12.53	0.90	9.49	35.83	53.24	46.76
		Low	22,040,140	65.80	17.61	1.53	2.97	20.39	36.48	63.52
**HSS**	Tertile	High	28,523,308	56.35	14.90	0.54	12.04	27.83	98.23	1.77
		Median	28,635,348	64.54	11.51	0.70	10.47	18.02	89.50	10.50
		Low	28,705,620	73.45	8.46	1.52	3.34	21.84	38.48	61.52
	Quartile	High	24,544,348	55.25	15.63	0.54	11.89	29.47	98.93	1.07
		2	18,296,366	62.58	12.88	0.55	12.37	16.69	96.32	3.68
		3	21,562,528	69.31	8.55	0.84	7.19	20.52	81.94	18.06
		Low	21,461,036	73.08	9.03	1.77	3.07	21.69	36.19	63.81

a. Percentage living in urban areas consisting of urbanized areas of 50,000 or more people and urban clusters of at least 2,500 and less than 50,000 people.

b. Percentage living in rural areas. “Rural” encompasses all population, housing, and territory not included within an urban area.

*SES* Socioeconomic status, *HSS* Health services supply, *N* Total number of SEER covered counties

In terms of direction and statistical significance, the indices performed similarly across tertiles and quartiles (Table 3). Consistent with previous findings, decreasing SES was associated with decreasing prostate and breast cancer incidence rates [13], primarily due to decreasing early-stage (i.e. localized/regional) prostate and breast cancer incidence rates. However, decreasing SES was statistically significantly associated with increasing advanced (distant) breast cancer and all stages (i.e. localized, regional, and distant) of lung cancer incidence rates. Further, increasing HSS was associated with increasing overall prostate and breast cancer incidence rates. Specifically, increasing HSS was statistically significantly associated with increasing early-stage prostate and breast cancer incidence rates. The HSS gradients detected in distant prostate and breast cancer incidence rates were not statistically significant. However, decreasing HSS was statistically significantly associated with increasing regional and distant lung cancer incidence rates.

**Table 3:** Distributions of 3-year age-adjusted cancer incidence rates, by SES and HSS, in 611 SEER^a^ counties.

Index	Classification scheme	Class	3-year age-adjusted incidence rates (per 100,000)^b^										
			Prostate cancer			Breast cancer				Lung and Bronchus cancer			
			All	Local/ Regional	Distant	All	Local	Regional	Distant	All	Local	Regional	Distant
SES index	Tertiles	High SES	167.97	152.37	8.85	162.19	106.67	41.91	10.70	66.19	13.48	16.00	33.45
		SES 2	162.74	147.65	10.21	153.03	96.49	43.81	11.60	75.29	14.03	17.06	39.48
		Low SES	154.55	137.72	10.49	142.06	86.14	41.18	13.50	99.11	18.35	23.48	50.47
		**% Change**^**c**^	**8.68**	**10.63**	**-**	**14.17**	**23.83**	**1.75**	**-20.76**	**-33.21**	**-26.52**	**-31.85**	**-33.72**
		***p-value***	*<0*.*01*	*<0*.*01*	*0*.*35*	*<0*.*01*	*<0*.*01*	*0*.*01*	*<0*.*01*	*<0*.*01*	*0*.*02*	*<0*.*01*	*<0*.*01*
	Quartiles	High SES	169.05	153.55	8.37	166.32	109.38	43.25	10.63	67.96	14.51	15.82	33.93
		SES 2	165.37	150.79	10.45	154.22	97.84	43.04	11.76	72.30	13.23	17.59	37.32
		SES 3	160.50	144.32	9.69	151.68	96.11	43.33	11.04	75.75	14.34	16.49	39.74
		Low SES	154.40	137.60	10.53	141.53	85.78	41.03	13.55	99.35	18.33	23.46	50.35
		**% Change**^**c**^	**9.49**	**11.59**	**-**	**17.51**	**27.51**	**5.42**	**-21.57**	**-31.59**	**-20.86**	**-32.56**	**-32.62**
		***p-value***	*<0*.*01*	*<0*.*01*	*0*.*40*	*<0*.*01*	*<0*.*01*	*<0*.*01*	*0*.*01*	*<0*.*01*	*<0*.*01*	*<0*.*01*	*<0*.*01*
HSS index	Tertiles	High HSS	183.95	164.49	9.32	162.80	102.60	44.77	11.84	71.32	14.57	16.46	35.85
		HSS 2	176.34	159.27	9.13	165.35	105.17	45.43	11.69	79.63	16.51	18.65	40.08
		Low HSS	154.96	138.91	10.39	144.09	88.82	41.28	11.39	91.26	16.83	21.49	46.66
		**% Change**^**c**^	**18.71**	**18.41**	**-**	**12.99**	**15.51**	**8.44**	**-**	**-21.85**	**-**	**-23.41**	**-23.16**
		***p-value***	*<0*.*01*	*<0*.*01*	*0*.*84*	*<0*.*01*	*<0*.*01*	*<0*.*01*	*0*.*38*	*<0*.*01*	*0*.*29*	*<0*.*01*	*<0*.*01*
	Quartiles	High HSS	185.80	164.92	9.40	163.21	102.56	44.34	12.26	73.02	14.94	16.94	36.21
		HSS 2	186.58	169.64	9.28	168.52	107.02	46.58	11.90	74.32	15.24	17.42	38.15
		HSS 3	171.49	155.58	8.78	159.51	102.01	43.72	11.03	83.25	17.17	19.50	41.76
		Low HSS	153.85	137.72	10.54	143.72	88.34	41.31	13.00	91.76	16.82	21.61	46.95
		**% Change**^**c**^	**20.77**	**19.75**	**-**	**13.56**	**16.10**	**7.33**	**-**	**-20.42**	**-**	**-21.62**	**-22.88**
		***p-value***	*<0*.*01*	*<0*.*01*	*0*.*57*	*<0*.*01*	*<0*.*01*	*<0*.*01*	*0*.*45*	*<0*.*01*	*0*.*42*	*<0*.*01*	*<0*.*01*

a. SEER data used to assess the gradients detected by SES and HSS measures included individuals at least 15 years of age, who lived in counties covered by 17 SEER registries at cancer diagnosis from 2010 to 2012. Alaska Natives, Kalawao County in Hawaii and unknown counties were excluded.

b. Rates are per 100,000 and age-adjusted to the 2000 US Standard Population (19 age groups).

c. % Change in incidence rates = ((High-Low category)/High category)X100

*SEER* Surveillance, Epidemiology, and End Results program, *SES* Socioeconomic status, *HSS* Health Services Supply.

The contribution of county-level SES and HSS to racial differences in cancer incidence varied by cancer site. Overall the prostate, breast, lung and bronchus cancer incidence rates among Whites and African Americans were higher than the rates among Asian/Pacific islanders. Decreasing SES and HSS were associated with decreasing prostate cancer incidence rates among Whites and increasing rates among Asian/Pacific islanders. Decreasing HSS was also statistically significantly associated with decreasing prostate cancer incidence rates among African Americans. Decreasing SES and HSS were both associated with increasing lung and bronchus cancer incidence rates among Whites, African Americans and Asian/Pacific islanders.

## Discussion

Consistent with previous studies [6, 7, 13, 43], we found that increasing county-level SES was associated with increasing prostate and breast cancer, and decreasing lung cancer incidence rates [6, 7, 13, 43]. Although several studies have explored the impact of individual health services characteristics such as physician density or hospitals on cancer incidence [23, 24, 44–46], a wide range of health care services are needed to foster early detection and better health outcomes in the population. In order to address this gap in the literature, we developed a novel HSS index capturing the availability of a wide range of health care facilities (physician offices, medical and diagnostic laboratories, general medical and surgical hospitals), physicians (primary care practitioners, public health and general preventive medicine practitioners) and non-physician health care providers (physician assistants, nurses, pharmacists and diagnostic radiologists) to assess the effects of area-level health services on cancer outcomes. The novel county-level HSS index showed that increasing availability of health care resources within counties was associated with increasing prostate and breast cancer and decreasing lung cancer incidence rates.

These patterns may be explained by the unequal distribution of socioeconomic resources in the society that can cause differential knowledge diffusion regarding prevention. The high prostate and breast cancer incidence rates in increasing SES and HSS counties were driven by increasing incidence rates of early-stage diagnoses. These results indicate that, greater awareness about prevention and higher screening rates in high SES and HSS counties may have led to earlier diagnosis of these cancers [13]. High lung and bronchus cancer incidence rates may have stemmed from high prevalence of smoking in low SES counties [17, 47], and lower access to health care resources that could influence behaviors relating to smoking cessation and screening in low HSS counties [22]. Therefore, information provided by both HSS and SES indices may prove useful in identifying areas for dissemination of cancer site-specific preventive knowledge and health services.

In this study, we revisited the measurement of county-level characteristics considering a richer, psychometrically induced approach [2]. Drawing on *a priori* criteria outlined by Oakes et al.,[2] we required that the indices we developed, 1) were constructed from valid, reliable and easily accessible data, 2) were responsive for analysis at the county-level, 3) had sound psychometric properties, 4) employed terms/concepts that policy-makers understand; and perhaps most importantly, 5) were practical and useful in applied public health and epidemiological surveys.

Area-level indices can be developed using both factor analysis and principal component analysis. While both methods may produce similar results, there are conceptual distinctions between the two approaches [48]. Principal component analysis is more suitable for data reduction [35, 48]. In this study, we used factor analysis, as the goal of the analysis was to arrive at a parsimonious representation of the associations among the measured area-level characteristics by identifying latent constructs underlying the SES and HSS variables [48].

This study has a few limitations that deserve mention. First, we combined different datasets to obtain information pertaining to health care facility and provider characteristics within counties, each of which probably has different sources of random error. Second, limited information on psychometric and distributional properties of previously developed indices restricted our ability to select an existing index to detect socioeconomic gradients at the county-level [5–8, 10, 22, 28]. Although there is no consensus definition of SES, the new index captures the main concepts of SES including employment, poverty, income and education. Housing, ownership and living crowdedness domains did not converge on to the same component during the modeling process. [13] Housing, ownership and living crowdedness tend to have different interpretations across rural and urban areas. For example, people in rural areas are more likely to own a house, own a car, and live in less crowded conditions. Therefore, exclusion of these measures and including only those measures that have the same relative meaning across geographic regions allowed the new index to have relatively simpler and uniform interpretations across the counties. Third, due to limited sample size we only created tertiles and quartiles for our analysis. However, previous studies have noted that tertiles may be more appropriate when the sample size is limited [13]. Fourth, county-level SES may not accurately represent individual socioeconomic characteristics, and equating differentials observed at the county-level to individual-level SES may lead to ecologic bias.[5, 49, 50] Therefore, caution should be exercised when comparing county-level vs. individual-level effects of SES on cancer outcomes. Finally, there may be other population-level social and behavioral factors contributing to these observed trends which were not explored in the current study [5].

## Conclusions

County-level SES and HSS indices are useful to assess geographic disparities in cancer incidence and stage at diagnosis. Further, these indices can be used in multi-level modeling studies to explain county-level variation in cancer outcomes.[51, 52] The gradients detected by county-level indices quantify cancer disparities attributable to county-level SES and HSS characteristics. Therefore, these measures may have the potential to provide useful tools to identify areas where resources could be allocated to reduce disparities in cancer outcomes. Future research could use these indices to examine the effects of county-level SES and HSS on population distributions of screening rates, smoking, crime, and environmental pollution to further explain geographic disparities in cancer outcomes.

## Supporting information

S1 Table Factor loadings and fit statistics for composite county-level SES indices constructed using SES measures identified in previous studies. a. White-collar occupations include management, professional, and related occupations.
b. Working class includes sales and office occupations, construction, extraction, and maintenance occupations, and production, transportation, and material moving occupations.
c. A weight of 16 was applied to the proportion of persons in the county with a college education (pc); 12 was applied to the proportion with a high school education (phs); and nine was applied to the proportion with less than a high school education (po). The average years of schooling in a given county, (E) = (16*pc) + (12*phs) + (9*po) [7].
d. Income disparity in year 2010 was defined as the 100×ratio of number of households with < $15,000 income to number of households with ≥$75,000 income.
e. Geographic level and US census years used to extract data in the original study to develop the index.
*N* Number of counties in 2000 US census, *SES* Socioeconomic status, *HH* Household, *N/A* Not Applicable.
(PDF)

S2 Table Factor loadings of the initial three-factor model developed for the SES index including 22 candidate variables extracted from the 2006–2010 American Community Survey 5-Year Estimates. a. White-collar occupations include management, professional, and related occupations.
b. A weight of 16 was applied to the proportion of persons in the county with a college education (pc); 12 was applied to the proportion with a high school education (phs); and nine was applied to the proportion with less than a high school education (po). The average years of schooling in a given county, E, is thus E = (16*pc) + (12*phs) + (9*po). [1]
c. Income disparity in year 2010 was defined as the 100×ratio of number of households with < $15,000 income to number of households with ≥$75,000 income.
*SES* Socioeconomic Status, *N* Number of counties, *HH* Household, *TLI* Tucker-Lewis Index.
(PDF)

## Abbreviations

ACS: American Community Survey
AHRF: Area Health Resource Files
CBP: County Business Patterns
FIPS: Federal Information and Processing Standards
HSS: Health Services Supply
ML: Maximum Likelihood
NCI: National Cancer Institute
SEER: Surveillance, Epidemiology, and End Results
SES: Socioeconomic Status
TLI: Tucker-Lewis Index
