# Supplementary material for: An ecological approach to monitor geographic disparities in cancer outcomes
Source: PLoS One. 2019 Jun 21;14(6):e0218712. doi: 10.1371/journal.pone.0218712 (PMC6588275; doi:10.1371/journal.pone.0218712)
Supplement: S1 Table — a. White-collar occupations include management, professional, and related occupations. b. Working class includes sales and office occupations, construction, extraction, and maintenance occupations, and production, transportation, and material moving occupations. c. A weight of 16 was applied to the proportion of persons in the county with a college education (pc); 12 was applied to the proportion with a high school education (phs); and nine was applied to the proportion with less than a high school education (po). The average years of schooling in a given county, (E) = (16*pc) + (12*phs) + (9*po) [7]. d. Income disparity in year 2010 was defined as the 100×ratio of number of households with < $15,000 income to number of households with ≥$75,000 income. e. Geographic level and US census years used to extract data in the original study to develop the index. N Number of counties in 2000 US census, SES Socioeconomic status, HH Household, N/A Not Applicable. (PDF) [file pone.0218712.s001.pdf]

S1 Table. Factor loadings and fit statistics for composite county-level SES indices constructed using SES measures identified in previous studies

SES domain	County-level SES measures	Singh [1] N=3,138)	Saldana-Ruiz [2] (N =3,138)	Yost [3] (N=3,138)	Krieger [4] (N=3,138)	Dayal [5] (N=3,138)	Rubin [6] (N=3,138)
Occupation	% white-collar occupations [a]	-0.308	0.421	N/A	N/A	N/A	0.375
	% working class [b]	N/A	N/A	- 0.162	-0.223	N/A	N/A
Education	% less than 9 years of education	0.631	-0.841	N/A	N/A	N/A	-0.838
	% aged ≥25 years and ≤12th grade of education	N/A	0.927	N/A	-0.809	N/A	0.898
	% aged ≥25 years with at least a high school diploma	0.253	N/A	N/A	N/A	-0.447	N/A
	% aged ≥25 years and ≥4 years of college	N/A	N/A	N/A	0.618	N/A	N/A
	Education index (weighted school years)[c]	N/A	N/A	0.683	N/A	N/A	N/A
Employment	% aged ≥16 years who are unemployed	0.297	N/A	-0.235	-0.426	N/A	N/A
	% aged ≥16 years who are employed	N/A	N/A	N/A	N/A	N/A	N/A
Poverty	% of persons below 150% of poverty line	0.889	N/A	-0.827	N/A	N/A	N/A
	% of families below poverty level	0.820	N/A	N/A	N/A	N/A	-0.807
	% of persons below poverty line	N/A	N/A	N/A	-0.879	-0.561	N/A
Income	Median family income	-0.949	N/A	N/A	N/A	N/A	N/A
	Income disparity [d]	0.799	N/A	N/A	N/A	N/A	N/A
	Median HH income	N/A	N/A	0.989	0.824	0.880	N/A
	% of total HH income in the area derived from interest, dividends, and net rent	N/A	N/A	N/A	0.630	N/A	N/A
Ownership	% home ownership	-0.468	N/A	N/A	0.528	N/A	N/A
	% car ownership	0.466	N/A	N/A	0.498	N/A	N/A
	% no telephone	0.683	N/A	N/A	-0.786	N/A	N/A
	% telephone access	N/A	0.668	N/A	N/A	N/A	0.729
Living crowdedness	% of HHs with more than one person per room	0.198	N/A	N/A	-0.371	N/A	N/A
	% of HHs with only one room	N/A	N/A	N/A	-0.088	N/A	N/A
	% of HHs without private plumbing	0.454	-0.214	N/A	-0.298	N/A	N/A
	% of HHs without kitchen	N/A	N/A	N/A	-0.060	N/A	N/A

SES domain	County-level SES measures	Singh [1] (N=3,138)	Saldana-Ruiz [2] (N =3,138)	Yost [3] (N=3,138)	Krieger [4] (N=3,138)	Dayal [5] (N=3,138)	Rubin [6] (N=3,138)
Living crowdedness Cont.	% single-parent HHs with children aged < 18 years	0.233	N/A	N/A	N/A	N/A	N/A
House	Median house value	-0.771	N/A	0.719	N/A	0.834	N/A
	Median rent	-0.840	N/A	0.849	N/A	0.959	N/A
	Median monthly mortgage	-0.866	N/A	N/A	N/A	N/A	N/A
	% with home worth ≥$300 k	N/A	N/A	N/A	0.362	N/A	N/A
**% of common variance explained**		**76.2 %**	**100%**	**84.8%**	**48.5%**	**92.5%**	**100%**
**TLI**		**0.816**	**0.800**	**0.530**	**0.287**	**0.516**	**0.729**
**Original study: area-level; US census years [e]**		Counties, Census tract and Zip code; 1970, 1990	County; 1970,1980, 1990, 2000	Census block groups in California; 1990	Census tract, block group, and zip code level for Massachusetts and Rhode Island; 1990	Census tract; 1970	County; 1970,1980, 1990, 2000

a. White-collar occupations include management, professional, and related occupations.
b. Working class includes sales and office occupations, construction, extraction, and maintenance occupations, and production, transportation, and material moving occupations.
c. A weight of 16 was applied to the proportion of persons in the county with a college education (pc); 12 was applied to the proportion with a high school education (phs) ; and nine was applied to the proportion with less than a high school education (po). The average years of schooling in a given county, (E) = (16*pc) + (12*phs) + (9*po). [7]
d. Income disparity in year 2010 was defined as the 100×ratio of number of households with < $15,000 income to number of households with ≥$75,000 income.
e. Geographic level and US census years used to extract data in the original study to develop the index.

[1] Singh GK (2003) Area deprivation and widening inequalities in US mortality, 1969-1998. Am J Public Health 93 (7):1137-1143
[2] Saldana-Ruiz N, Clouston SA, Rubin MS, Colen CG, Link BG (2013) Fundamental causes of colorectal cancer mortality in the United States: understanding the importance of socioeconomic status in creating inequality in mortality. American journal of public health 103 (1):99-104
[3] Yost K, Perkins C, Cohen R, Morris C, Wright W (2001) Socioeconomic status and breast cancer incidence in California for different race/ethnic groups. Cancer Causes Control 12 (8):703-711
[4] Krieger N, Chen JT, Waterman PD, Soobader MJ, Subramanian SV, Carson R (2002) Geocoding and monitoring of US socioeconomic inequalities in mortality and cancer incidence: does the choice of area-based measure and geographic level matter?: the Public Health Disparities Geocoding Project. Am J Epidemiol 156 (5):471-482
[5] Dayal HH, Power RN, Chiu C (1982) Race and socio-economic status in survival from breast cancer. Journal of chronic diseases 35 (8):675-683
[6] Rubin MS, Clouston S, Link BG (2014) A fundamental cause approach to the study of disparities in lung cancer and pancreatic cancer mortality in the United States. Soc Sci Med 100:54-61. doi:10.1016/j.socscimed.2013.10.026
[7] Liu L, Deapen D, Bernstein L. Socioeconomic status and cancers of the female breast and reproductive organs: a comparison across racial/ethnic populations in Los Angeles County, California (United States). Cancer Causes & Control. 1998;9(4):369-80.

N Number of counties in 2000 US census, *SES* Socioeconomic status, *HH* Household, *N/A* Not Applicable
