# Supplementary material for: An ecological approach to monitor geographic disparities in cancer outcomes
Source: PLoS One. 2019 Jun 21;14(6):e0218712. doi: 10.1371/journal.pone.0218712 (PMC6588275; doi:10.1371/journal.pone.0218712)
Supplement: S2 Table — a. White-collar occupations include management, professional, and related occupations. b. A weight of 16 was applied to the proportion of persons in the county with a college education (pc); 12 was applied to the proportion with a high school education (phs); and nine was applied to the proportion with less than a high school education (po). The average years of schooling in a given county, E, is thus E = (16*pc) + (12*phs) + (9*po). [1] c. Income disparity in year 2010 was defined as the 100×ratio of number of households with < $15,000 income to number of households with ≥$75,000 income. SES Socioeconomic Status, N Number of counties, HH Household, TLI Tucker-Lewis Index. (PDF) [file pone.0218712.s002.pdf]

S2 Table. Factor loadings of the initial three-factor model developed for the SES index including 22 candidate variables extracted from the 2006-2010 American Community Survey 5-Year Estimates

SES domain	County-level SES measures	Factor loadings (N=3,138)		
Occupation	% white-collar occupations[a]	0.584	0.532	-0.038
Education	% less than 9 years of education	-0.801	-0.243	0.090
	% aged ≥25 years and ≤12th grade of education	-0.881	-0.312	0.223
	% aged ≥25 years and ≥ 4 years of college	0.766	0.443	0.184
	Education index (weighted school years)[b]	0.879	0.368	-0.032
Employed	% aged ≥16 years who are employed	0.767	-0.152	-0.081
Poverty	% of persons below 150 % of poverty line	-0.910	0.376	0.116
	% of families below poverty level	-0.894	0.318	0.214
	% of persons below poverty line	-0.877	0.391	0.232
Income	Median family income	0.918	-0.221	0.228
	Income disparity[c]	-0.906	0.238	-0.268
	Median HH income	0.897	-0.300	0.266
	% of total HH income in the area derived from interest, dividends, and net rent	0.685	-0.070	0.315
Ownership	% home ownership	0.521	-0.250	-0.117
	% car ownership	0.577	-0.047	-0.326
	% no telephone	-0.815	-0.016	0.083
Living crowdedness	% of HHs w/more than one person per room	-0.291	0.207	0.582
	% of HHs w/o private plumbing	-0.560	-0.013	0.129
House	Median house value	0.696	-0.104	0.551
	Median rent	0.715	-0.073	0.565
	Median monthly mortgage	0.730	-0.089	0.591
	% with home worth ≥ $300 k	0.499	0.081	0.540
**% of common variance explained**		***80.1%***	***14.8%***	***5.1%***
**TLI**		**0.791**		

a. White-collar occupations include management, professional, and related occupations.

b. A weight of 16 was applied to the proportion of persons in the county with a college education (pc); 12 was applied to the proportion with a high school education (phs) ; and nine was applied to the proportion with less than a high school education (po). The average years of schooling in a given county, E, is thus E = (16*pc) + (12*phs) + (9*po). [1]
c. Income disparity in year 2010 was defined as the 100×ratio of number of households with < $15,000 income to number of households with ≥$75,000 income.

[1] Liu L, Deapen D, Bernstein L. Socioeconomic status and cancers of the female breast and reproductive organs: a comparison across racial/ethnic populations in Los Angeles County, California (United States). Cancer Causes & Control. 1998;9(4):369-80.

SES Socioeconomic Status, *N* Number of counties, *HH* Household, *TLI* Tucker-Lewis Index
